# Breast cancer molecular subtypes and survival in a hospital-based sample in Puerto Rico

**DOI:** 10.1002/cam4.78

**Published:** 2013-04-18

**Authors:** Ana Patricia Ortiz, Orquidea Frías, Javier Pérez, Fernando Cabanillas, Lisa Martínez, Carola Sánchez, David E Capó-Ramos, Carmen González-Keelan, Edna Mora, Erick Suárez

**Affiliations:** 1 Cancer Control and Population Sciences Program University of Puerto Rico Comprehensive Cancer CenterSan Juan, Puerto Rico; 2 Department of Biostatistics and Epidemiology, Graduate School of Public Health, Medical Sciences Campus, University of Puerto RicoSan Juan, Puerto Rico; 3 Puerto Rico Central Cancer Registry, University of Puerto Rico Comprehensive Cancer CenterSan Juan, Puerto Rico; 4 Cancer Center, Hospital Auxilio MutuoSan Juan, Puerto Rico; 5 School of Medicine, Medical Sciences Campus, University of Puerto RicoSan Juan, Puerto Rico; 6 National Center for Health Statistics, Centers for Disease Control & PreventionMaryland; 7 Department of Pathology, School of Medicine, Medical Sciences Campus, University of Puerto RicoSan Juan, Puerto Rico; 8 Department of Surgery, School of Medicine, Medical Sciences Campus, University of Puerto RicoSan Juan, Puerto Rico

**Keywords:** Breast cancer, Hispanic, Puerto Rico, subtypes, survival

## Abstract

Information on the impact of hormone receptor status subtypes in breast cancer (BC) prognosis is still limited for Hispanics. We aimed to evaluate the association of BC molecular subtypes and other clinical factors with survival in a hospital-based female population of BC cases in Puerto Rico. We analyzed 663 cases of invasive BC diagnosed between 2002 and 2005. Information on HER-2/neu (HER-2) overexpression, estrogen (ER), and progesterone (PR) receptor status and clinical characteristics were retrieved from hospitals cancer registries and record review. Survival probabilities by covariates of interest were described using the Kaplan–Meier estimators. Cox proportional hazards models were employed to assess factors associated with risk of BC death. Overall, 17.3% of BC cases were triple-negative (TN), 61.8% were Luminal-A, 13.3% were Luminal-B, and 7.5% were HER-2 overexpressed. In the multivariate Cox model, among patients with localized stage, women with TN BC had higher risk of death (adjusted hazard ratio [HR]: 2.57, 95% confidence interval [CI]: 1.29–5.12) as compared to those with Luminal-A status, after adjusting for age at diagnosis. In addition, among women with regional/distant stage at diagnosis, those with TN BC (HR: 5.48, 95% CI: 2.63–11.47) and those HER-2+, including HER-2 overexpressed and Luminal-B, (HR: 2.73, 95% CI:1.30–5.75) had a higher mortality. This is the most comprehensive epidemiological study to date on the impact of hormone receptor expression subtypes in BC survival in Puerto Rico. Consistent to results in other populations, the TN subtype and HER-2+ tumors were associated with decreased survival.

## Introduction

Breast cancer (BC) is the most common female malignancy in Puerto Rico and the United States (US) [1, 2]. Variations in BC occurrence and outcome exist by geographic regions and ethnic background [2–4]. The risk of developing BC is increasing faster in Puerto Rico than among non-Hispanic whites (NHW) in the US [5]. Despite lower incidence rates in Puerto Rico, these are increasing [1, 6], although they have remained stable for Hispanics and NHW [2, 6].

Breast cancer is a multifaceted disease comprising distinct biological subtypes with diverse etiology, therapeutic indications, and clinical outcomes [7, 8]. Human epidermal growth factor 2 (HER-2), estrogen (ER), and progesterone (PR) receptors are the three most common diagnostic markers that drive the clinical management of BC patients. Tumor-cell expression of these receptors has implications for disease progression as well as specific therapeutic interventions [9]. HER-2 overexpression predicts response to treatment, and is associated with more aggressive cancers and worse clinical outcomes, including survival [10]. Patients who are negative for ER and PR do not respond to established endocrine therapy and have poorer prognoses when compared with their ER and PR+ counterparts [11].

Biomarker phenotypes can be grouped into four tumor categories with different histological characteristics: Luminal-A is ER+ and/or PR+/HER-2−, Luminal-B is ER+ and/or PR+/HER-2+, HER-2 overexpressed is ER−/PR−/HER-2+, and triple-negative (TN) is ER−/PR−/HER-2− [10, 12]. The TN subtype is linked to aggressive cancers, metastasis [13], negative clinical outcomes, and is also frequently observed in BRCA1-related BC [14, 15].

Breast cancer development and mortality are also affected by demographic factors, including age [16, 17], race and ethnicity [4, 18, 19], and socio-economic status (SES) [20]. Although African Americans have lower incidence of BC, they have higher mortality from the disease than NHW [21, 22], a disparity that is accounted, in part, to the higher prevalence of TN BC in this group [23–25]. Several studies show that Hispanics have a lower incidence of BC but a higher BC-related mortality rate compared with NHW [6, 26], a finding also observed for Puerto Ricans [6]. Significant differences in the genetics and biology of BC in Hispanics, including a higher incidence of TN BCs [25–27] have been described as a significant contributor to their higher mortality [27]. Nonetheless, these results have not been consistent across studies [28]. A study in Puerto Rico suggests that the clinical outcome in Hispanic women with TN BC is more likely explained by SES and access to services rather than biological/genetic differences [29].

Despite availability of information on the impact of BC molecular subtypes in disease prognosis, these data are still limited for Hispanics. To further understand BC disparities in this group, we evaluated for the first time the association between BC molecular subtypes and other clinical factors with survival among Puerto Rico female BC cases.

## Methods

The study population consisted of invasive BC cases diagnosed from 2002 to 2005 at the *I. González Martínez Oncologic Hospital* and the *Auxilio Mutuo Hospital*. During this period, 1072 incident cases of BC were identified. We reviewed medical records and pathology reports and extracted data from the cancer registries of both hospitals to collect information on HER-2 (corroborated by the presence of an immunohistochemistry [IHC] pathology report), ER and PR status of study participants, as well as data on age at diagnosis, disease grade, stage, death, and other clinical covariates. Patients with missing information on HER-2 (*n* = 359) or ER and/or PR status (*n* = 10), and those with inconclusive HER-2 results (*n* = 40) were excluded. In total, we analyzed data from 663 female patients at the *I. González Martínez Hospital* (*n* = 318) and the *Auxilio Mutuo Hospital* (*n* = 345). This study was approved by the Institutional Review Boards of the University of Puerto Rico Medical Sciences Campus and of both hospitals.

Information on tumor receptor expression of cases was obtained from record review of IHC pathological analyses. According to the staining intensity, the pathologist categorized HER-2 status as positive (IHC score = 3+), negative (IHC score = 0, 1+), or equivocal (inconclusive IHC score = 2+, inconclusive cases were excluded from the analyses); ER and PR status as positive or negative. Using this information, patients were categorized into four tumor subtypes, according to their tumor marker status as: (1) TN (HER-2−/ER−/PR−); (2) Luminal-A (HER-2−/ER and/or PR+); (3) Luminal-B (HER-2+/ER and/or PR+); and (4) HER-2 overexpressing (HER-2+, ER−, PR−) [12]. Data on age at diagnosis (<50 years, ≥50 years [cutoff used to differentiate early onset BC]) [30, 31], tumor histology (lobular, ductal, other), size (<2 cm, ≥2 cm), and grade (I, well differentiated; II, moderately well differentiated; III, poorly differentiated; and IV, undifferentiated) were also recorded. Information on stage at diagnosis (localized, regional, distant) was defined according to Surveillance Epidemiology and End Results (SEER) Summary Staging criteria. For cases diagnosed between 2002 and 2005, the SEER Summary Staging 2000 codes were used [32]. Lymph node metastasis (yes, no) at time of diagnosis was assessed, as well as information on vital status at last contact. Information on clinical characteristics of patients and of their date of last contact and vital status were corroborated with the Puerto Rico Central Cancer Registry.

We used descriptive statistics to characterize the study sample. We compared the characteristics associated with BC tumor subtypes among BC patients using chi-square distributions. Survival analysis was performed among patients diagnosed from 2002 to 2005, with the maximum follow-up date being 31 December 2009. Median follow-up time of patients was 24.3 months (minimum: 0.10 months, maximum: 83.2 months).

We described covariates' survival probabilities using the Kaplan–Meier estimators. The survival curves between categories of BC were compared using the Wilcoxon test in order to weight with the population at risk at the most recent time of follow-up; the number of population at risk with long time of follow-up was very small so the survival curves dramatically changed the slope. However, we also assessed the comparison of the survival curves with the log-rank test and the results were similar [33]. We used Cox proportional hazards models to estimate the magnitude of association between BC subtype and risk of death, after adjusting for stage and age at diagnosis through hazard ratio (HR) with 95% confidence interval (CI). We assessed interaction terms within the Cox model using the likelihood ratio test (LRT) and the proportional hazard with the Schoenfeld residual method [33]. Data analysis was performed using Stata 12.

## Results

### Study population

Median age of diagnosis of patients was 57.0 years (percentile 25: 48.1, percentile 75: 68.0). Overall, 17.3% of cases were TN, 61.8% were Luminal-A, 13.3% Luminal-B, and 7.5% were HER-2 overexpressed. Significant differences in the clinical characteristics studied were observed by BC subtypes (*P* < 0.05) (Table 1). Comparison of study individuals (*n* = 663), with those excluded because of missing information for molecular subtype (*n* = 409) showed that these groups did not differ in any of the clinical characteristics under study (*P* > 0.05, data not shown).

**Table 1:** Clinical characteristics of the study population, overall and by tumor subtype (*n* = 663)

Characteristics	Triple-negative HER-2−, ER−, PR− (*n* = 115, 17.3%)	Luminal-A HER-2−, ER and/or PR+ (*n* = 410, 61.8%)	Luminal-B HER-2+, ER and/or PR+ (*n* = 88, 13.3%)	Her-2 overexpressed HER-2+, ER−, PR− (*n* = 50, 7.5%)	*P*-value
	*n* (%)	*n* (%)	*n* (%)	*n* (%)	*n* (%)
Age at diagnosis					
<50 years	41 (35.6)	108 (26.3)	37 (42.0)	26 (52.0)	<0.001
≥50 years	74 (64.4)	302 (73.7)	51 (58.0)	24 (48.0)	
Age at diagnosis (*μ* ± SD)	55.8 ± 1.2	59.9 ± 0.7	56.3 ± 1.5	52.6 ± 2.2	<0.001*
Tumor histology (*n* = 658)					
Invasive lobular	4 (3.5)	94 (23.1)	13 (14.9)	1 (2.0)	<0.001**
Invasive ductal	101 (88.6)	281 (69.0)	70 (80.5)	48 (96.0)	
Other	9 (7.9)	32 (7.9)	4 (4.6)	1 (2.0)	
Tumor grade (*n* = 566)					
I – Well differentiated	5 (5.0)	75 (21.9)	5 (6.8)	1 (2.2)	<0.001**
II – Moderately differentiated	29 (28.7)	180 (52.5)	37 (48.7)	18 (39.1)	
III – Poorly differentiated	58 (57.4)	79 (23.0)	25 (32.9)	25 (54.3)	
IV – Undifferentiated aggressive	9 (8.9)	9 (2.6)	9 (11.8)	2 (4.3)	
Tumor size (*n* = 544)					
<2 cm	31 (30.4)	189 (55.4)	30 (44.1)	10 (30.3)	<0.001
≥2 cm	71 (69.6)	152 (44.6)	38 (55.9)	13 (69.7)	
LN metastasis (*n* = 552)					
Negative	51 (57.9)	233 (66.9)	40 (55.6)	20 (45.5)	0.014
Positive	37 (42.1)	115 (33.1)	32 (44.4)	24 (54.5)	
Tumor staging (*n* = 612)					
Localized	62 (59.6)	248 (65.1)	43 (55.1)	24 (49.0)	0.078
Regional/distant	42 (40.4)	133 (34.9)	35 (44.9)	25 (51.0)	
Hospital				0.335	
Oncologic	61 (53.0)	187 (45.6)	42 (47.7)	28 (56.0)	0.335
Auxilio mutuo	54 (47.0)	223 (54.4)	46 (52.3)	22 (44.0)	

Table 1 shows significant differences in the characteristics of the study population, by tumor subtypes.

* Oneway Anova for comparing means.

** Fishers exact test *P*-value.

### Five-year survival

The overall 5-year survival for the entire sample was 71.2%. When stratified by tumor subtype, women with Luminal-A BC had the highest 5-year survival (80.2%) and those TN had the lowest (47.7%) (Table 2).

**Table 2:** Five-year survival, overall, and by tumor subtypes

Tumor subtype	5-years survival	95%CI
Triple-negative (HER-2−, ER−, PR−)	47.7%	32.2%–61.6%
Luminal-A (HER-2−, ER and/or PR +)	80.2%	72.4%–85.9%
Luminal-B (HER-2+, ER and/or PR+)	63.0%	42.2%–78.1%
HER-2 overexpressing (HER-2+, ER−, PR−)	72.3%	53.6%–84.5%
Overall survival	71.2%	64.9%–76.5%

Table 2 shows that women with TN BC had the lowest 5-year survival.

### Factors associated with risk of death

For Cox proportional hazards modeling, women with Luminal-B and HER-2 overexpressing disease were combined into a category called “HER-2+,” given reduced sample size of women with HER-2 overexpressing disease (*n* = 50) and given that survival curves significantly overlapped between these groups (data not shown). When using these categorizations, the proportional hazard assumption was satisfied (*P* = 0.80), and we proceeded with bivariate analysis. In Kaplan–Meier survival curves, significant differences in the survival functions of BC subtypes were observed (*P* < 0.0001). In Cox regression models, women with TN BC (HR: 3.02, 95% CI = 1.94–4.70) and those HER-2+ (HR: 1.79, 95% CI = 1.12–2.87) had higher risk of death as compared to those with Luminal-A disease. Other factors associated with risk of death in bivariate analysis (*P* < 0.05) included younger age at diagnosis and advanced stage; no differences were observed by tumor histology or by source hospital (*P* > 0.05) (Table 3).

**Table 3:** Hazard ratio (HR) to assess the factors associated with risk of death (*n* = 663)

Characteristics	HR crude (95% CI)
Breast cancer subtype	
Luminal-A (HER-2−, ER and/or PR+)	1.00
Triple-negative (HER-2−, ER−, PR−)	3.02 (1.94–4.70)
HER-2+ (HER-2+, ER and/or PR±)	1.79 (1.12–2.87)
Age at diagnosis	
<50 years	1.57 (1.02–2.43)
≥50 years	1.00
Tumor staging (*n* = 573)	
Localized	1.00
Regional/distant	2.35 (1.58–3.48)
Tumor histology (*n* = 658)	
Invasive lobular	1.00
Invasive ductal	1.68 (0.90–3.16)
Other	2.46 (1.02–5.94)
Tumor grade (*n* = 566)	
I – Well differentiated	1.00
II – Moderately differentiated	3.86 (1.20–12.46)
III – Poorly differentiated	5.71 (1.77–18.46)
IV – Undifferentiated aggressive	2.39 (0.40–14.33)
Tumor size (*n* = 544)	
<2 cm	1.00
≥2 cm	1.70 (1.06–2.70)
LN metastasis (*n* = 552)	
Negative	1.00
Positive	2.12 (1.37–3.27)
Hospital	
Oncologic hospital	1.00
Auxilio mutuo	1.18 (0.80–1.75)

Table 3 presents the results from the crude Cox proportional hazards models showing that HER-2+ women and those with TN BC have increased risk of death as compared to those Luminal-A. Other factors associated to risk of death in bivariate analyses included age at diagnosis <50 years, regional/distant stage, and lymph node metastasis.

**Figure 1:**
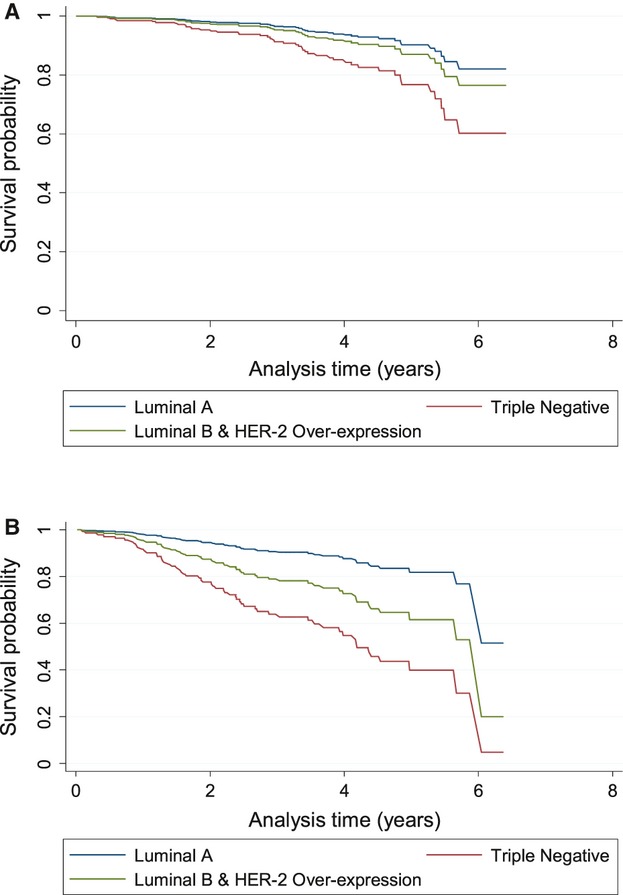
(A) Age-adjusted survival curves for women with localized stage at time of diagnosis, by breast cancer subtype. (B) Age-adjusted survival curves for women with regional/distant stage at time of diagnosis, by breast cancer subtype.

The initial Cox multivariate model considered the association between BC subtype and risk of death, after adjusting for stage and age at diagnosis. Given the presence of significant interaction terms in the Cox model (LRT *P* < 0.001), the survival analysis was performed separately in each stage, adjusting for age at diagnosis. In the Cox model among patients with localized stage, women with TN BC had higher risk of death (HR: 2.57, 95% CI = 1.29–5.12) as compared to those with Luminal-A status (Table 4). Meanwhile, among women with regional/distant staging, those with TN BC (HR: 5.48, 95% CI: 2.63–11.47) and HER-2+ (HR: 2.73, 95% CI = 1.30–5.75) had increased risk of death as compared to those with Luminal-A disease (Fig. 1A and B).

**Table 4:** Hazard ratio (HR) to assess the factors associated with risk of death*

	Stage	
	Localized (*n* = 377)	Regional/distant (*n* = 196)
Characteristics	HR adjusted (95% CI)	
Breast cancer subtype		
Luminal-A (HER-2−, ER and/or PR +)	1.00	1.00
Triple-negative (HER-2−, ER−, PR−)	2.57 (1.29–5.12)	5.48 (11.47)
HER-2+ (HER-2+, ER and/or PR±)	1.36 (0.60–3.09)	2.73 (1.26–5.75)
Age at diagnosis		
<50 years	2.34 (1.06–5.13)	1.85 (0.97–3.56)
≥50 years	1.00	1.00

* Proportional hazards assumption was evaluated and found satisfied (staging localized: *P* = 0.6849; staging regional/distant: *P* = 0.8345) after stratification.

Table 4 shows that in both staging categories, women with TN BC had higher risk of death as compared to those with Luminal-A status, after adjusting for age at diagnosis.

## Discussion

This study describes the influence of BC molecular subtypes on risk of death in a Hispanic population. Although the distribution of molecular subtypes in our clinic-based study may not be representative of Puerto Rico, our estimates have similarities and contrasts with those from other US populations. Our TN status (17.3%) results are similar to those for NHW (16.7%) and lower than African Americans estimates (24.6%) [34], although higher than those described for Hispanics (10.7%) in another study [12]. Our estimates for HER-2 positivity were similar to previous data for Hispanics (Luminal-B: 13.3% vs. 14.2% and HER-2 overexpressing: 7.5% vs. 6.6%) [12, 35]. Meanwhile, our results for TN status are somewhat similar to those overall (13.1%) and for Hispanics (17.6%) in a big US cohort [36], while our estimates of HER-2 positivity are similar to theirs (Luminal-B: 15.5% and HER-2 overexpressed: 7.2%) and specifically to their Hispanic population (Luminal-B: 16.4% and HER-2 overexpressed: 9.7%) [36].

Our study reveals differences in the clinical characteristics of patients with different molecular subtypes, with a higher proportion of women with TN status and HER-2+ having larger tumor size, advanced stage at diagnosis and lymph node metastasis. Also, the highest proportion of cases <50 years was observed among those HER-2+ and those with TN BC, as compared to those with Luminal-A BC. This is consistent with previous studies that have documented younger age at diagnosis, larger tumor size, lymphovascular invasion and higher grade at diagnosis among TN BC cases [37–40] and those HER-2+ [37, 41, 42] as compared to those with Luminal-A disease.

Our overall 5-year survival estimates (71.2%) are lower than those of US [28, 36] women and other populations [43, 44]. Meanwhile, similar to previous studies, women with Luminal-A BC (80.2%) had the highest 5-year survival [36] and those with TN disease had the lowest 5-year survival (47.7%) [43, 45]. Nonetheless, some studies [28, 36] have also observed lowest survival among patients with HER-2 overexpression, similar to that of TN cases, a pattern that was not observed in our study and that could not be assessed in multivariate analyses given the small proportion of HER-2 overexpressed tumors.

The clinical correlates of BC survival in this population are also similar to those described previously in the US. In stratified analysis, our results showed that among women with regional/distant stage at diagnosis, those with HER-2+ had more than twofold higher risk of death, when compared to those in the Luminal-A group. Nonetheless, among those with localized disease, this excess risk of death was not observed for women HER-2+. HER-2 receptor overexpression has been clearly linked to a greater risk of tumor recurrence after initial remission [32, 45], as well as diminished survival [34, 46]. The inclusion of trastuzumab in the initial treatment of BC patients with HER-2+ tumors has changed the recurrence rate and survival of these patients. In Puerto Rico, Herceptin had been available since its initial launch in 1998. It was approved for treatment of metastatic HER-2+ BC. This intervention most probably affected the survival of some of these patients. However, in 2006, trastuzumab was also approved by the Food and Drug Administration for adjuvant treatment of BC patients. As a result, the patients included in this study were not treated in the adjuvant setting. Future studies evaluating the outcome of treatment of HER-2+ patients will determine whether adjuvant trastuzumab affects patient survival. While we do not have complete data on initial treatment regimen, it is possible that the known risk of adverse outcomes of HER-2 overexpressing tumors, coupled with the availability of effective therapeutic agents to treat these HER-2+ tumors [47] may be causing physicians to treat these tumors more quickly and aggressively than others. This may lead to improved outcomes for these patients, including greater survival, and may partly explain why HER-2 overexpression showed no association with mortality in our sample of women with localized disease.

Meanwhile, consistent with previous studies [28, 48, 49] among women with both localized and regional/distant stage at diagnosis, those with TN BC had 3–5 times increased risk of death as compared to those with Luminal-A disease. Poor prognosis of patients with TN BC was also seen in another study in Puerto Rico [29]. Also, as in previous studies [16, 20, 28, 34, 50], patients with age at diagnosis <50 years had increased risk of death.

Similar with results in other populations, the TN subtype was associated with decreased survival time [24, 25] and the Luminal-A subtype was associated with the greatest survival [28]. Given that studies suggest that the TN subtype is more common among Hispanics than among NHW [26], these results highlight the importance of HER-2 and hormone receptor screening in this population.

Future studies should aim to further elucidate other socio-economic and cultural factors that may impact BC survival in Puerto Rico. This is important given that 41.5% of Puerto Ricans live at or below poverty line [51] and given the significant financial burden of a BC diagnosis [3]. It is also pertinent to evaluate the effect of lifestyles and other clinical factors on BC survival in this population, and their potential interactions with BC molecular subtypes.

Our study may be limited by selection bias, as 38.2% of BC cases were excluded because of missing information on HER-2 or ER/PR receptors. However, study individuals did not differ from those excluded in any of the clinical characteristics studied. The absence of this information in medical records is of concern given that routine HER-2 and ER/PR receptor screening recommendations for treatment decision were established prior to the study period [52, 53]. Also, our hospital-based data may not be representative of all BC cases in Puerto Rico.

This is the most comprehensive study to date on the impact of BC molecular subtypes on BC survival in Puerto Rico. Consistent with other populations, the TN subtype was associated with decreased survival, even when stratified by tumor staging. Furthermore, HER-2+ cases with localized disease also had higher risk of death as compared to those with Luminal-A BC. Given the observed biological differences in BC between racial/ethnic groups [17] and the recognized need for additional studies on BC subtypes [54], our study contributes to this area by revealing the survival disadvantage of women with these molecular subtypes in a population of Hispanic origin. Considering that targeted treatments exist for these tumor subtypes, our results highlight the need for more effective management of TN and HER-2+ cancers in Puerto Rico, including phenotype specific BC control strategies.
